# Quality of life in adolescents with recently diagnosed childhood-onset systemic lupus erythematosus

**DOI:** 10.1186/1546-0096-10-S1-A19

**Published:** 2012-07-13

**Authors:** Sarah Tuck, Tricia Williams, Earl D Silverman, Deborah M Levy

**Affiliations:** 1 The Hospital for Sick Children, Toronto, ON, Canada

## Purpose

The unpredictable disease course of childhood-onset systemic lupus erythematosus (cSLE) and its neuropsychiatric and cognitive symptoms can negatively impact quality of life at a time of major social, psychological and cognitive development. Quality of life (QoL) is multidimensional construct that describes a person’s self perception of physical, emotional, social and school/vocational functioning. Our purpose was to examine four health-related QoL domains (i.e., physical, emotional, social and school functioning) and depressive symptoms in adolescents with recently diagnosed cSLE from a patient and parent perspective, and to evaluate for associations between the QoL domains and depression.

## Methods

A preliminary sample of twenty cSLE subjects (Mean Age 14.9 ± 2.3 years, 75% female) and their parents completed standardized questionnaires assessing quality of life (PedsQL) and depression (Children’s Depression Inventory, CDI) as part of an observational research study of neurocognitive function in cSLE patients within 18 months from diagnosis of cSLE. Correlations and repeated measures ANOVA were used to examine parent-youth agreement and associations between QoL domains and depressive symptoms. PedsQL scaled scores were calculated for each domain, with higher scores representing greater health-related QoL (range 0 - 100).

## Results

Both cSLE subjects and their parents reported lower QoL scores in the adolescent’s physical, emotional and school functioning as compared to social functioning, which remained relatively less affected (mean scaled scores 67.0, 61.7, 63.9 and 82.6 respectively, *p*<.001). Mean CDI scores were within the normal range, with 3 subjects (15%) scoring in the depressive symptom range. Depressive symptoms were strongly correlated with the emotional difficulties domain for both the cSLE self-report (*r* = -0.73, *p*<.01) and for the parent report (*r* = -0.84, *p*<.01). Similarly, depressive symptoms were correlated with the physical challenges domain for both cSLE self-report (*r* = -0.61, *p*< .05) and parent report (*r* = -0.50, *p*<.05), and their reported problems at school (*r* = -0.61, *p*< .01).

## Conclusion

In this preliminary report we found that cSLE affects not only physical functioning domain of QoL early on, but also the domains of emotional and school functioning, within months of diagnosis. Not surprisingly, symptoms of depression were associated with multiple aspects of quality of life, including emotional, school and physical functioning. Further study will examine for associations of QoL with neurocognitive function.

## Disclosure

Sarah Tuck: None; Tricia Williams: None; Earl D. Silverman: None; Deborah M. Levy: None.

